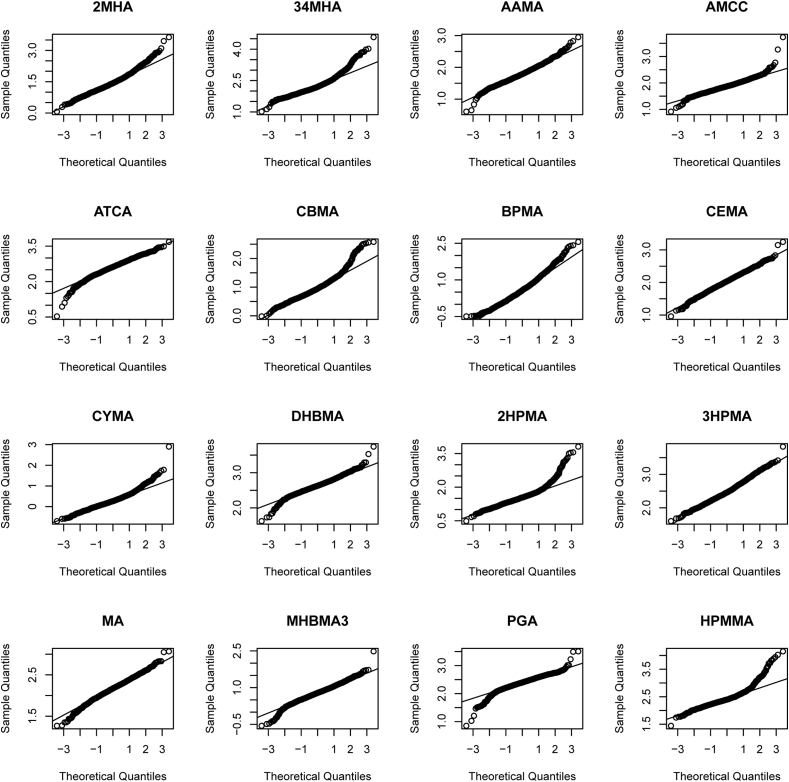# The urinary metabolites of volatile organic compounds and asthma in young children: NHANES 2011–2018

**DOI:** 10.1016/j.heliyon.2024.e24199

**Published:** 2024-01-23

**Authors:** Yixiao Xiong, Xin Liu, Tao Li

**Affiliations:** a Department of Anesthesiology, West China Hospital, Sichuan University, Sichuan, 610041, China; b Laboratory of Mitochondria and Metabolism, National-Local Joint Engineering Research Centre of Translational Medicine of Anesthesiology, West China Hospital, Sichuan University, Sichuan, 610041, China

**Keywords:** Volatile organic compounds, Metabolites of volatile organic compounds, Childhood asthma, NHANES

## Abstract

The vast majority of volatile organic compounds (VOCs) are of biological origin and do not affect human health, while some VOCs or their oxidation products can damage the respiratory system, nervous system, digestive system and blood system after long-term inhalation by humans. There is limited evidence regarding the association of VOCs exposure with childhood asthma. In this study, we examined the associations between metabolites of VOCs (mVOCs) in urine and childhood asthma. We included a total of 1542 children aged 3–12 years who had information on urinary mVOCs, asthma and essential covariates in the current analyses. After controlling for covariates, we used logistic regression to assess the association between urinary mVOCs and childhood asthma. Then, we examined effect measure modification by child age, gender, race/ethnicity and serum cotinine. 2-Methylhippuric acid (xylene metabolites) (OR: 1.14; 95 % CI: 0.87, 1.59), N-acetyl-S-(benzyl)-l-cysteine (toluene metabolites) (OR: 1.15 95 % CI: 0.76, 1.71), N-acetyl-S-(2-carboxyethyl)-l-cysteine (acrolein metabolites) (OR: 1.09; 95 % CI: 0.61, 1.75), N-acetyl-S-(3-hydroxypropyl)-l-cysteine (acrolein metabolites) (OR: 1.10; 95 % CI: 0.66, 1.80), and N-acetyl-S-(3-hydroxypropyl-1-methyl)-l-cysteine (crotonaldehyde metabolites) (OR: 1.18; 95 % CI: 0.68, 2.01) were weakly associated with the prevalence of asthma in children. Among female children, 2MHA (2-methylhippuric acid) in urine was significantly associated with the prevalence of asthma (OR: 1.81 95 % CI: 1.07, 3.05). At the same time, BMA (N-acetyl-S-(benzyl)-l-cysteine) was significantly associated with the prevalence of asthma in non-Hispanic White (OR:2.09 95 % CI: 0.91, 4.66) and Black (OR:1.90 95 % CI: 0.96, 3.71) children. We found that gender modified the associations between urinary 2MHA and the odds of asthma (interaction term p value = 0.03). Therefore, exposure to VOCs and the development of childhood asthma remains controversial, and the interpretation of these results needs to be treated with caution and should be confirmed in future studies.Therefore, exposure to VOCs and the development of childhood asthma remains controversial, and the interpretation of these results needs to be treated with caution and should be confirmed in future studies.

## 1 Introduction

Childhood asthma, an extremely prevalent disease, poses a significant burden on children's health [[1], [2], [3]]. Asthma cannot be cured, but its symptoms can be controlled [4]. The probability of having asthma in children aged 5–14 years is 6.6 %. Although the proportion of adults with asthma (8.4 %) is significantly greater than that of children (5.8 %), a large proportion of it is due to childhood asthma that is carried over [5]. The identification of modifiable risk factors for asthma (especially in children) could cause the disease and reduce the associated morbidity [6]. Early exposure of children to environmental chemicals is a potentially modifiable risk factor that may have an adverse effect on the immune system, which may increase the risk of asthma [[7], [8], [9]].

Most of the VOCs in the Earth's atmosphere are emitted by plants. However, man-made VOCs have a greater impact on the health of all human organ systems [10]. Man-made VOCs are synthetic chemicals that are mainly from industrial waste gas, automobile exhaust and photochemical pollution from fuel combustion and transportation [11]. As emissions of man-made VOCs rise year by year, their concentrations in the atmosphere are increasing [12]. The most important pathways for the absorption of VOCs are inhalation of environmental gases and dietary intake [3]. Long-term inhalation of man-made VOCs can cause direct damage to the respiratory system first, followed by the nervous system, digestive system and blood system [11,13]. Directly measuring the concentration of VOCs in the air and other environmental media is not included as a primary detection method because of its lack of stability and accuracy. The metabolites of volatile organic compounds in urine have a longer physiological half-life and are present in the body for a longer period of time than those measured in blood, while not requiring invasive sampling, making mVOCs a more meaningful biomarker of long-term VOCs exposure [14,15].

Most previous studies have focused on the association of blood VOCs with asthma and exhaled VOCs as biomarkers for asthma diagnosis, while few studies have examined the association of urinary mVOCs, an indicator of long-term exposure to airborne VOCs, with asthma prevalence. Mercapturic acid is produced by the reaction of VOCs with glutathione in the body and excreted in the urine. For the detection of VOCs in urine, the main test is their metabolites, which are the corresponding mercapturic acids [16]. The mVOCs that can be detected in urine originate from 16 organics, mainly including N-Acetyl-S-(2-carboxyethyl)-l-cysteine (CEMA) and N-Acetyl-S-(3-hydroxypropyl)-l-cysteine (3HPMA) (acrolein metabolites), N-Acetyl-S-(2-carbamoylethyl)-l-cysteine (AAMA) and N-Acetyl-S-(2-carbamoyl-2-hydroxyethyl)-lcysteine (GAMA) (acrylamide metabolites), N-Acetyl-S-(2-cyanoethyl)-l-cysteine (CYMA) (acrylonitrile metabolite), N-Acetyl-S-(2-hydroxyethyl)-l-cysteine (HEMA) (acrylonitrile, vinyl chloride, ethylene oxide metabolite), N-Acetyl-S-(n-propyl)-l-cysteine (BPMA) (1-bromopropane metabolite), N-Acetyl-S-(3,4-dihydroxybutyl)-l-cysteine (DHBMA), N-Acetyl-S-(1-hydroxymethyl-2-propenyl)-lcysteine (MHBMA1), N-Acetyl-S-(2-hydroxy-3-butenyl)-l-cysteine (MHBMA2) and N-Acetyl-S-(4-hydroxy-2-buten-1-yl)-lcysteine (MHBMA3) (1,3-butadiene metabolites), N-Acetyl-S-(3-hydroxypropyl-1-methyl)-lcysteine (HPMMA) (crotonaldehyde metabolite), 2-Aminothiazoline-4-carboxylic acid (ATCA) (cyanide metabolite), N-Acetyl-S-(N-methylcarbamoyl)-l-cysteine (AMCC) (N,N-Dimethylformamide metabolite) and Phenylglyoxylic acid (PGA) (ethylbenzene and styrene metabolite), N-Acetyl-S-(2-hydroxypropyl)-l-cysteine (2HPMA) (Propylene oxide metabolite), N-Acetyl-S-(1-phenyl-2-hydroxyethyl)-lcysteine + N-Acetyl-S-(2-phenyl-2-hydroxyethyl)-lcysteine (PHEMA) and Mandelic acid (MA) (styrene metabolites) and N–N-Acetyl-S-(benzyl)-l-cysteine (BMA) (toluene), 2-Methylhippuric acid (2MHA) and 3-Methylhippuric acid + 4-Methylhippuric acid (34 MHA) (xylene metabolites) [16]. To identify environmental chemicals that potentially cause asthma in children exposed at an early age, we investigated the association between individual and multiple VOCs coexposure and the development of asthma in US children, as indicated by the levels of mVOCs in urine. We also conducted stratified analyses by gender, age, and race to explore the relationship between urinary mVOCs concentration and childhood asthma prevalence.

## 2 Methods

### 2.1 Study population

**Fig. 1:**
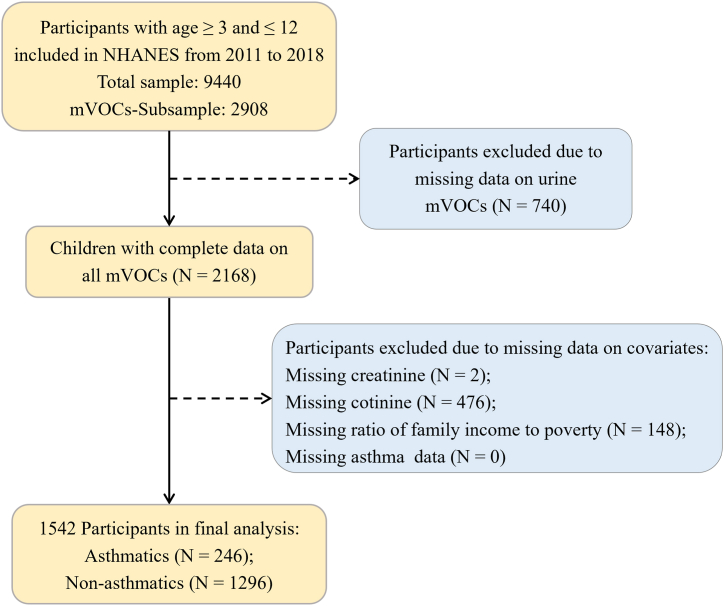
Selection of the study population.

NHANES is a series of national cross-sectional, complex multistage, representative surveys and aimed to assess the health and nutritional status of noninstitutionalized people in the United States. All survey procedures and data content were allowed to be published only after written informed consent was obtained from all participants and approved by the National Center for Health Statistics (NCHS) Ethic Review Board. All surveys and measurements were completed in a single visit with the subject's consent. For our analysis, we included children aged 3–12 years who had completed all urinary mVOCs measurements. Then, we excluded participants with missing covariate data. Finally, a total of 1542 children were enrolled in this study. The participant selection is displayed in Fig. 1. The specific sources of NHANES data covered by the study are listed in the supplementary materials (Table S1).

### 2.2 Laboratory methods

In the absence of a requirement for precollection fasting, participants were asked to randomly urinate into a sample cup, after which the samples were stored at −20 °C. In this study, sixteen urinary mVOCs (2MHA, 34MA, AAMA, ATCA, BMA, BPMA, CEMA, CYMA, DHBMA, 2HPMA, 3HPMA, MA, MHBMA3, PGA and HPMMA) were quantified in children aged 3–12 years participating in the NHANES 2011–2018 survey cycle (n = 1542). The concentrations of five mVOCs in urine samples were determined using a precise analytical chemistry method [16]. When the mVOCs detection result is below the lower limit of detection (LLOD), LLOD/√2 is used as the input filler value to replace LLOD. Then, for more representative data and robust outcomes, we excluded urinary mVOCs with detection values below the LLOD exceeding 50 % of the total participants from the analysis (Table S2). Detailed descriptions of the analytical methods and quality assurance/quality control procedures have been disclosed on the NHANES website [17].

### 2.3 Outcome and covariables

Asthma diagnosis and age of asthma were determined by the data file of medical conditions (MCQ) (question: “has a doctor or other health professional ever told you that study participant has asthma?”). We considered adjusting for variables associated with childhood urinary mVOCs concentrations and asthma diagnosis or believed to confound this relationship. Age (in years), gender (male or female), race/ethnicity (non-Hispanic White, non-Hispanic Black, Hispanic, and other races) and the level of poverty (ratio of income to poverty) were extracted from the demographic variables file (DEMO). The cut-off points for poverty were defined by the NHANES survey variable, which was calculated as a ratio of individual/family income to poverty with a range from 0 to 5. According to the Department of Health and Human Services recommendations, we defined values from <1.36 as “poor”, 1.36–1.86 as “nearly poor”, and values from ≥1.86 as “not poor”.

### 2.4 Statistical analyses

All data analyses were performed using Stata 17.0 and R version 4.2.1. We used published subsample weights (WTSA2YR) designed for a one-third subset of the entire survey for all effects and variance estimates to produce estimates representative of the U.S. population, as recommended by NHANES. Considering the concentration and dilution of urine, we log10-transformed the mVOCs levels of creatinine-corrected urine and plotted a Q‒Q plot to confirm that mVOCs followed a roughly normal distribution after the logarithmic transformation (Fig. S1). Then, normalization was performed to allow for subsequent analysis. We calculated geometric means (GM) and interquartile range (IQR) for all sixteen mVOCs and by prevalence of asthma. Weighted Pearson correlation was used to assess the extent to which all urinary mVOCs were interrelated.

Logistic regression was used to assess the extent to which urinary concentrations of mVOCs were associated with the prevalence of asthma in children. In the unadjusted model, we estimated the odds ratio of ln-transformed urinary mVOCs concentrations per standard deviation (SD) increase for asthma by logistic regression. In the adjusted model, we adjusted for child age (continuous), gender (binary), race/ethnicity (categorical), poverty to income ratio (continuous), ln-transformed serum cotinine concentration (continuous) and estimated the odds ratio of ln-transformed creatinine-corrected mVOCs concentrations in current asthma per standard deviation (SD) increase in urine. We used Wald tests to detect differences in model coefficients for the mVOCs variable between different levels of modifiers. We also performed stratified regression analyses by age group, gender, and race/ethnicity. Given that mVOCs were measured in children with asthma after asthma diagnosis, we performed additional sensitivity analyses. We tested whether the difference between current age and age at diagnosis of asthma predicts urinary mVOCs concentrations in patients with asthma by Pearson correlation. To assess the overall association of exposure to mixed mVOCs and to identify the most toxic substances in mVOCs, we used weighted quantile sum (WQS) regression analyses, assuming linear and additivity effects, to create empirically weighted indices of chemical substances based on the quantiles and to use the indices as individual exposure terms in the regression models18.We divided the data into training (40 %) and validation (60 %) datasets. We created positive and negative WQS indices for all the results to account for the potentially different directions of influence among the urinary VOC metabolites.

## 3 Results

Of the children between 3 and 12 years old in the NHANES 2011–2018 cycle, 1542 participants were included in our analyses after excluding participants with missing data. Among the samples analyzed, there were 773 (weighted 52 %) male children, 397 (weighted 14 %) were Black, and 479 (weighted 25 %) were Hispanic. There were 246 (weighted 17 %) study participants with doctor-diagnosed asthma (Table 1).

**Table 1:** Demographic characteristics for children aged 3–12 years old in NHANES 2011–2018 (n = 1542).

Characteristics	n	Weighted %
Overall	1542	
Asthmatics	246	17
Non-asthmatics	1296	83
Age, years (mean ± sd)	7.6 ± 0.1	
3–6	591	24
7–12	951	76
Gender		
Male	773	52
Female	769	48
Race/Ethnicity		
Hispanic	479	25
White, non-Hispanic	417	51
Black, non-Hispanic	397	14
Other	249	10
Poverty:Income^a^		
Poor	729	36
Nearly poor	197	13
Not poor	616	51

a Ratio of family income to poverty.

**Fig. 2:**
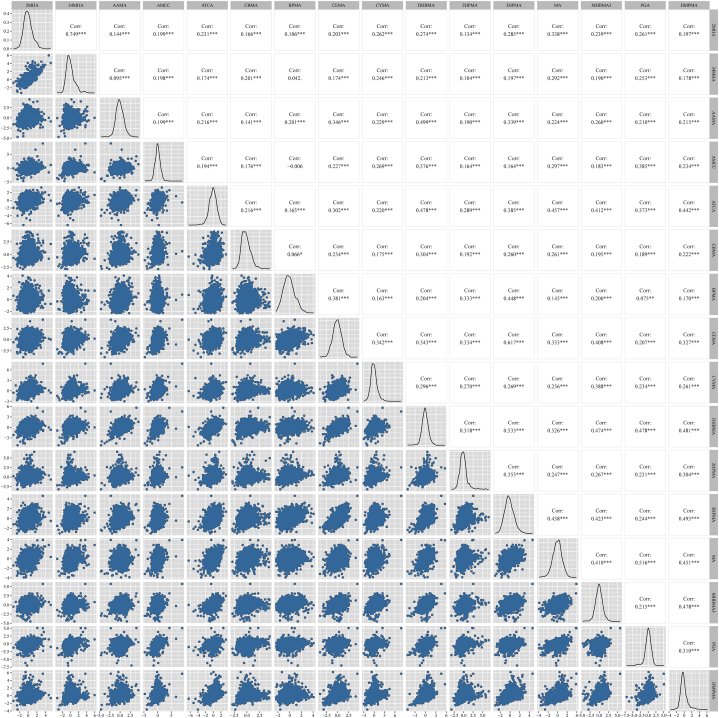
Weighted Pearson correlation coefficients for the (standardized) ln-transformed of creatinine-corrected mVOCs concentrations (ng/mg Cr) in children age 3–12 years (n = 1542): NHANES (2011–2018). On the diagonal is a histogram of the distribution of ln-transformed creatinine-corrected mVOCs concentrations; above the diagonal is the correlation coefficient; and below the diagonal is the scatter plot. *p < 0.05, **p < 0.01, ***p < 0.001.

The parent compounds, GM and IQR of urinary creatinine-corrected mVOCs concentrations (ng/mg Cr) are listed (Table 2). We observed that certain creatinine-corrected mVOCs within the asthma group were significantly higher than those in the non-asthma group, including xylene metabolites (2MHA and 34MHA), acrolein metabolites (CEMA and 3HPMA) and toluene metabolite (BMA). Then, Pearson correlation matrix plots were used to identify the degree of correlation between ln-transformed creatinine-corrected mVOCs (Fig. 2). With the exception of strong correlations for metabolites from the same parent compound source, the urinary creatinine-corrected 1,3-butadiene metabolite (DHBMA) was positively correlated with acrolein metabolites (CEMA and 3HPMA) (r = 0.543 and r = 0.533), and the 1,3-butadiene metabolite (DHBMA) was positively correlated with metabolites of styrene (MA) (r = 0.526). The correlations between the remaining mVOCs were moderate, weak, or nonsignificant.

**Table 2:** Weighted GM (IQR) creatinine-corrected urinary mVOCs concentrations (ng/mg Cr) in children aged 3–12 years, NHANES 2011–2018.

Urinary mVOCs	Parent Compounds	Total (n = 1542)	Asthmatics (n = 246)	Non-asthmatics (n = 1296)
2MHA	Xylene	29.2 (15.3, 49.8)	29.9 (15.3, 50.3)	28.8 (15.3, 48.2)
34MHA	Xylene	182.6 (103.9, 280.9)	185.3 (97.5, 260.8)	182.1 (105.1, 287.4)
AAMA	Acrylamide	60.8 (42.5, 91.0)	59.5 (41.6, 86.2)	61.1 (42.7, 92.0)
AMCC	Acrylamide	76.6 (56.3, 100.0)	73.6 (54.5, 90.1)	77.3 (56.6, 102.0)
ATCA	Cyanide	368.9 (259.1, 661.3)	336.3 (235.3, 624.2)	375.8 (265.4, 665.5)
BMA	Toluene	8.7 (5.7, 15.0)	8.9 (5.7, 15.2)	8.6 (5.1, 14.9)
BPMA	1-Bromopropane	3.7 (1.9, 7.7)	3.5 (1.8, 8.2)	3.8 (1.9, 7.6)
CEMA	Acrolein	100.5 (71.9, 165.6)	102.0 (72.4, 108.1)	99.7 (70.4, 161.6)
CYMA	Acrylonitrile	1.9 (1.2, 3.0)	1.9 (1.2, 2.8)	1.9 (1.2, 3.1)
DHBMA	1,3-Butadiene	414.9 (326.2, 565.6)	385.2 (292.6, 531.6)	421.0 (337.2, 572.9)
2HPMA	Propylene oxide	35.2 (23.2, 51.6)	35.2 (21.2, 51.1)	35.2 (23.7, 51.7)
3HPMA	Acrolein	276.7 (192.7, 475.5)	278.3 (194.2, 476.1)	276.3 (187.9, 460.3)
MA	Styrene	141.0 (105.3, 204.1)	128.5 (93.8, 188.9)	143.7 (108.4, 206.5)
MHBMA3	1,3-Butadiene	5.8 (3.9, 9.1)	5.6 (3.6, 8.9)	5.8 (4.0, 9.2)
PGA	Ethylbenzene, styrene	242.4 (185.6, 249.2)	223.8 (172.3, 305.7)	246.3 (188.4, 339.0)
HPMMA	Crotonaldehyde	260.0 (191.7, 343.2)	255.9 (179.4, 339.0)	260.8 (194.1, 362.4)

Abbreviations: NHANES: National Health and Nutrition Examination Survey; GM: geometric mean; IQR: interquartile range; mVOCs: metabolites of volatile organic compounds; 2MHA: 2-methylhippuric acid; 34MHA: 3-and 4-methylhippuric acid; AAMA: N-acetyl-S-(2-carbamoylethyl)-l-cysteine; AMCC: N-acetyl-S-(N-methylcarbamoyl)-l-cysteine; ATCA: 2-aminothiazoline-4-carboxylic acid; BMA: N-acetyl-S-(benzyl)-l-cysteine; BPMA: N-acetyl-S-(n-propyl)-l-cysteine; CEMA: N-acetyl-S-(2-carboxyethyl)-l-cysteine; CYMA: N-acetyl-S-(2-cyanoethyl)-l-cysteine; DHBMA: N-acetyl-S-(3:4-dihydroxybutyl)-l-cysteine; 2HPMA: N-acetyl-S-(2-hydroxypropyl)-l-cysteine; 3HPMA: N-acetyl-S-(3-hydroxypropyl)-l-cysteine; MA: mandelic acid; MHBMA3: N-acetyl-S-(4-hydroxy-2-butenyl)-l-cysteine; PGA: phenylglyoxylic acid; HPMMA: N-acetyl-S-(3-hydroxypropyl-1-methyl)-l-cysteine.

After covariate adjustment, the asthma risk increased by 15 %, 9 %, 10 %, and 18 % in response to each SD increase in ln-transformed creatinine-corrected BMA (OR: 1.15; 95 % CI: 0.76, 1.71), CEMA (OR: 1.09; 95 % CI: 0.66, 1.75), 3HPMA (OR: 1.10; 95 % CI: 0.66, 1.80), and HPMMA (OR: 1.18; 95 % CI: 0.68, 2.01), respectively (Table 2). At the same time, in a multi-pollutant logistic regression analysis, we found that ln-transformed creatinine-corrected 2MHA (OR: 1.68; 95 % CI: 0.99, 2.88) was positively associated with the development of asthma in children. However, creatinine-corrected DHBMA (OR: 0.42; 95 % CI: 0.19, 0.96) and MA (OR: 0.48; 95 % CI: 0.25, 0.92) were significantly and negatively associated with the development of childhood asthma (Table 3). Gender did modify associations between ln-transformed creatinine-corrected 2MHA concentrations and odds of asthma (gender-by-2MHA interaction p value = 0.03) (Table 4). Specifically, for every SD increase in ln-transformed creatinine-corrected 2MHA concentrations, female children had 81 % increased odds of asthma (OR: 1.81; 95 % CI: 1.07, 3.05) compared with male children (OR: 0.83; 95 % CI: 0.52, 1.31). Meanwhile, Age modified associations between ln-transformed creatinine-corrected AMCC concentrations and odds of asthma (Age-by-AMCC interaction p value = 0.04), but ln-transformed creatinine-corrected AMCC negatively associated with the development of childhood asthma aged 3–6. Race/ethnicity did not modify the association between ln-transformed creatinine-corrected urinary mVOCs concentrations (interaction term p values = 0.12–1.00) (Table 2). At the same time, we observed a significant positive correlation between creatinine-corrected 2MHA (OR: 1.69; 95 % CI: 0.93, 3.06) and BMA (OR: 1.90; 95 % CI: 0.96, 3.71) and the development of current asthma in non-Hispanic black children. The odds of having developed asthma in non-Hispanic white children were positively associated with creatinine-corrected metabolites of toluene (BMA, OR: 2.09; 95 % CI: 0.90, 4.66). However, creatinine-corrected DHBMA was significantly and negatively associated with asthma prevalence in children aged 7–12 years (OR: 0.37; 95 % CI: 0.14, 0.96). The difference in current age and age of asthma diagnoses was not correlated with ln-transformed creatinine-corrected mVOCs concentrations (Table S2).

**Table 3:** Unadjusted and adjusted ORs for parent-reported asthma diagnosis among children aged 3–12 years per SD increase in ln-transformed urinary creatinine-corrected mVOCs concentrations (n = 1559): NHANES (2011–2018).

Urinary mVOCs	Unadjusted	Multipollutant^a^	Adjusted^b^	Multipollutant
	OR (95 % CI)	OR (95 % CI)	OR (95 % CI)	OR (95 % CI)
2MHA	1.05 (0.92, 1.66)	1.46 (0.87, 2.48)	1.14 (0.81, 1.59)	**1.68 (0.99, 2.88)**
34MHA	0.87 (0.60, 1.24)	0.78 (0.44, 1.34)	0.94 (0.65, 1.35)	0.69 (0.39, 1.20)
AAMA	0.73 (0.43, 1.25)	1.19 (0.64, 2.23)	0.89 (0.50, 1.55)	1.17 (0.61, 2.21)
AMCC	**0.46 (0.23, 0.90)**	0.75 (0.34, 1.63)	0.65 (0.31, 1.32)	0.86 (0.38, 1.89)
ATCA	0.71 (0.47, 1.07)	1.10 (0.65, 1.90)	1.01 (0.61, 1.67)	1.27 (0.72, 2.27)
BMA	1.01 (0.88, 1.64)	1.15 (0.75, 1.73)	1.15 (0.76, 1.71)	1.30 (0.85, 1.97)
BPMA	0.95 (0.71, 1.26)	0.91 (0.64, 1.27)	0.94 (0.71, 1.26)	0.88 (0.62, 1.23)
CEMA	0.98 (0.52, 1.42)	1.54 (0.76, 3.12)	1.09 (0.61, 1.75)	1.45 (0.71, 2.95)
CYMA	0.88 (0.58, 1.32)	1.07 (0.66, 1.71)	0.78 (0.49, 1.26)	0.84 (0.48, 1.45)
DHBMA	**0.27 (0.14, 0.54)**	**0.19 (0.06, 0.61)**	**0.42 (0.19, 0.96)**	**0.30 (0.09, 0.97)**
2HPMA	0.76 (0.50, 1.16)	0.89 (0.54, 1.42)	0.90 (0.58, 1.36)	0.92 (0.55, 1.48)
3HPMA	0.98(0.53, 1.37)	1.37 (0.66, 2.80)	1.10 (0.66, 1.80)	1.32 (0.63, 2.73)
MA	**0.36 (0.20, 0.65)**	**0.42 (0.19, 0.94)**	**0.48 (0.25, 0.92)**	**0.43 (0.19, 0.98)**
MHBMA3	0.70 (0.44, 1.10)	0.89 (0.49, 1.61)	0.83 (0.51, 1.34)	0.85 (0.47, 1.58)
PGA	**0.42 (0.23, 0.76)**	0.86 (0.40, 1.94)	0.56 (0.30, 1.10)	0.89 (0.41, 2.03)
HPMMA	1.02 (0.86, 1.77)	1.44 (0.75, 2.67)	1.18 (0.68, 2.01)	1.68 (0.88, 3.11)

Abbreviations: OR: odds ratio; CI: confidence interval; mVOCs: metabolites of volatile organic compounds; NHANES: National Health and Nutrition Examination Survey; SD: standard deviation. 2MHA: 2-methylhippuric acid; 34MHA: 3-and 4-methylhippuric acid; AAMA: N-acetyl-S-(2-carbamoylethyl)-l-cysteine; AMCC: N-acetyl-S-(N-methylcarbamoyl)-l-cysteine; ATCA: 2-aminothiazoline-4-carboxylic acid; BMA: N-acetyl-S-(benzyl)-l-cysteine; BPMA: N-acetyl-S-(n-propyl)-l-cysteine; CEMA: N-acetyl-S-(2-carboxyethyl)-l-cysteine; CYMA: N-acetyl-S-(2-cyanoethyl)-l-cysteine; DHBMA: N-acetyl-S-(3:4-dihydroxybutyl)-l-cysteine; 2HPMA: N-acetyl-S-(2-hydroxypropyl)-l-cysteine; 3HPMA: N-acetyl-S-(3-hydroxypropyl)-l-cysteine; MA: mandelic acid; MHBMA3: N-acetyl-S-(4-hydroxy-2-butenyl)-l-cysteine; atcaPGA: phenylglyoxylic acid; HPMMA: N-acetyl-S-(3-hydroxypropyl-1-methyl)-l-cysteine.

Bold data indicates significant at p < 0.05.

a Regression models adjusted for other urinary VOC concentrations.

b Regression models adjusted for gender, age, race/ethnicity, poverty to income ratio, and serum cotinine.

**Table 4:** Stratified regression analyses by age group, gender, and race/ethnicity (n = 1559), NHANES 2011–2018.

Urinary mVOCs	Age			Gender			Race/Ethnicity				
	Age 3-6	Age 7-12	*p*	Male	Female	*p*	Hispanic	White, non-Hispanic	Black, non-Hispanic	Other	*p*
2MHA	1.62 (0.89, 2.91)	0.96 (0.64, 1.44)	0.36	0.83 (0.52, 1.31)	**1.81 (1.07, 3.05)**	**0.03**	0.91 (0.45, 1.81)	0.82 (0.40, 1.62)	**1.69 (0.93, 3.06)**	1.52 (0.68, 3.16)	0.12
34MHA	0.68 (0.32, 1.35)	1.05 (0.68, 1.61)	0.31	0.80 (0.48, 1.28)	1.24 (0.70, 2.16)	0.15	0.81 (0.36, 1.70)	0.85 (0.38, 1.79)	1.06 (0.57, 1.90)	1.12 (0.44, 2.75)	0.32
AAMA	1.21 (0.46, 3.19)	0.72 (0.36, 1.44)	0.96	0.74 (0.34, 1.58)	1.08 (0.47, 2.51)	0.37	0.53 (0.18, 1.58)	1.06 (0.28, 3.42)	0.88 (0.32, 2.36)	1.37 (0.42, 4.46)	0.29
AMCC	**0.26 (0.07, 0.95)**	0.92 (0.39, 2.11)	**0.04**	0.61 (0.23, 1.62)	0.70 (0.24, 2.61)	0.71	0.63 (0.15, 2.52)	0.94 (0.21, 3.68)	0.35 (0.08, 1.40)	1.21 (0.22, 4.73)	0.79
ATCA	1.14 (0.46, 2.97)	0.81 (0.47, 1.41)	0.47	0.85 (0.43, 1.66)	1.20 (0.56, 2.63)	0.39	1.13 (0.43, 3.13)	0.96 (0.32, 2.95)	1.05 (0.45, 2.52)	0.96 (0.30, 3.19)	0.88
BMA	0.99 (0.46, 2.00)	1.15 (0.70, 1.85)	0.52	1.08 (0.62, 1.84)	1.25 (0.67, 2.27)	0.62	0.45 (0.17, 1.13)	**2.09 (0.91, 4.66)**	**1.90 (0.96, 3.71)**	0.46 (0.16, 1.22)	0.47
BPMA	0.90 (0.51, 1.56)	0.97 (0.69, 1.36)	0.59	1.10 (0.75, 1.60)	0.77 (0.48, 1.19)	0.24	0.83 (0.44, 1.54)	0.63 (0.31,1.23)	1.14 (0.71, 1.82)	1.04 (0.55, 1.92)	0.38
CEMA	0.77 (0.30, 2.07)	1.08 (0.58, 2.01)	0.65	0.86 (0.43, 1.72)	1.29 (0.57, 2.88)	0.33	0.79 (0.29, 2.16)	0.95 (0.31, 2.84)	0.82 (0.31, 2.22)	1.54 (0.47, 4.10)	0.32
CYMA	0.58 (0.23, 1.38)	0.80 (0.46, 1.40)	0.38	0.77 (0.40, 1.47)	0.80 (0.39, 1.57)	0.29	0.43 (0.15, 1.22)	1.79 (0.75, 4.18)	0.49 (0.21, 1.15)	0.95 (0.29, 2.99)	0.64
DHBMA	0.45 (0.11, 1.88)	**0.37 (0.14, 0.96)**	0.54	0.44 (0.03, 1.35)	0.37 (0.11, 1.24)	0.99	0.24 (0.05, 1.27)	0.93 (0.16, 5.43)	0.31 (0.07, 1.40)	0.73 (0.14, 4.03)	0.32
2HPMA	0.57 (0.22, 1.30)	1.00 (0.60, 1.63)	0.29	0.93 (0.51, 1.61)	0.83 (0.41, 1.57)	0.96	0.76 (0.32, 1.69)	1.54 (0.60, 3.65)	0.87 (0.37, 1.93)	1.05 (0.21, 1.51)	1.00
3HPMA	1.31 (0.54, 3.17)	0.94 (0.51, 1.72)	0.99	1.27 (0.65, 2.48)	0.84 (0.38, 1.83)	0.50	0.91 (0.33, 2.40)	1.60 (0.53, 4.69)	1.25 (0.49, 3.16)	0.74 (0.25, 2.20)	0.95
MA	0.50 (0.17, 1.52)	**0.42 (0.19, 0.92)**	0.99	0.55 (0.23, 1.32)	0.38 (0.14, 1.02)	0.62	**0.21 (0.06, 0.78)**	0.86 (0.23, 3.35)	0.44 (0.14, 1.42)	0.81 (0.17, 4.02)	0.12
MHBMA3	0.59 (0.24, 1.47)	0.83 (0.48, 1.46)	0.91	0.79 (0.42, 1.49)	0.83 (0.39, 1.74)	0.83	0.52 (0.21, 1.27)	1.54 (0.53, 4.32)	0.82 (0.31, 2.13)	0.98 (0.38, 2.66)	0.32
PGA	0.56 (0.19, 1.80)	0.50 (0.23, 1.08)	0.47	0.52 (0.24, 1.15)	0.62 (0.20, 2.0)	0.72	0.45 (0.15, 1.43)	1.02 (0.24, 4.68)	0.66 (0.19, 2.43)	0.42 (0.09, 2.21)	0.57
HPMMA	0.96 (0.36, 2.30)	1.10 (0.56, 2.07)	0.59	0.87 (0.41, 1.74)	1.86 (0.76, 3.32)	0.28	1.95 (0.67, 5.23)	1.69 (0.54, 4.76)	1.30 (0.42, 3.82)	0.46 (0.11, 1.54)	0.31

Data are presented as ORs (95 % CI). Abbreviations: NHANES: National Health and Nutrition Examination Survey; OR: odds ratio; CI: confidence interval; 2MHA: 2-methylhippuric acid; 34MHA: 3-and 4-methylhippuric acid; AAMA: N-acetyl-S-(2-carbamoylethyl)-l-cysteine; AMCC: N-acetyl-S-(N-methylcarbamoyl)-l-cysteine; ATCA: 2-aminothiazoline-4-carboxylic acid; BMA: N-acetyl-S-(benzyl)-l-cysteine; BPMA: N-acetyl-S-(n-propyl)-l-cysteine; CEMA: N-acetyl-S-(2-carboxyethyl)-l-cysteine; CYMA: N-acetyl-S-(2-cyanoethyl)-l-cysteine; DHBMA: N-acetyl-S-(3:4-dihydroxybutyl)-l-cysteine; 2HPMA: N-acetyl-S-(2-hydroxypropyl)-l-cysteine; 3HPMA: N-acetyl-S-(3-hydroxypropyl)-l-cysteine; MA: mandelic acid; MHBMA3: N-acetyl-S-(4-hydroxy-2-butenyl)-l-cysteine; PGA: phenylglyoxylic acid; HPMMA: N-acetyl-S-(3-hydroxypropyl-1-methyl)-l-cysteine.

*P*: age, gender, and race/ethnicity-mVOCs interaction p-value.

Bold data indicates significant at p < 0.05.

**Fig. 3:**
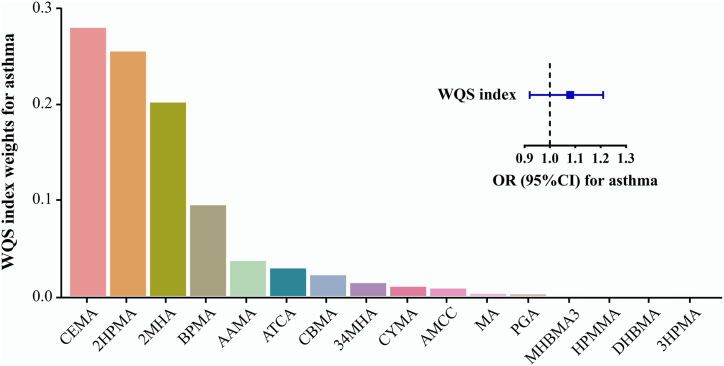
WQS model regression index weights for childhood asthma when the association in WQS analyses was assumed in positive direction in total participants. Weights estimates were adjusted for age (continuous), gender (binary), race/ethnicity (categorical), poverty to income ratio (continuous), and ln-transformed serum cotinine concentration (continuous).

The WQS index was positively associated with childhood asthma (OR:1.08; 95 % CI: 0.92, 0.21). CEMA (metabolites of acrolein), 2HPMA (propylene oxide metabolite) and 2MHA (xylene metabolite) are the main co-builders of the positive association, with weights of 28 %, 25 % and 20 %, respectively (Fig. 3).

## 4 Discussion

This cross-sectional study is the first to investigate the associations of urinary metabolites of VOCs and asthma in children aged 3–12 years. We observed a positive correlation between creatinine-corrected BMA, CEMA,3HPMA and HPMMA and the development of asthma in children. Our findings also indicate that gender significantly modified the associations between ln-transformed creatinine-corrected 2MHA (xylene metabolite) concentrations and asthma diagnosis. In the stratified analyses, BMA (toluene metabolite) was significantly associated with the development of asthma in non-Hispanic White and Black children. The results of WQS analysis suggested that metabolite of acrolein (CEMA), metabolite of propylene oxide (2HPMA) metabolite of toluene (BMA) are the major contributors to the positive association.

Non-occupational exposure to VOCs is primarily caused by cigarette smoke, which consists of thousands of toxic chemicals and carcinogenic substances [18,19]. However, there is still relatively little research on the accumulation of organic compounds in the body and the processes that affect human metabolism. Recently, a cross-sectional study from NHANES reported that urinary volatile organic compound metabolites of acrolein (CEMA, 3HPMA) and crotonaldehyde (HPMMA) were associated with reduced lung function in 1342 adults >18 years of age, which is consistent with our finding of the major roles of CEMA and 3HPMA in the positive association of mVOCs with asthma in children [20]. However, the available evidence of childhood asthma induced by VOCs exposure is still contradictory. A case‒control study reported that children with an average age of 8 years had higher levels of VOCs exposure in their urine than healthy children, and mVOCs (2MHA, 34MHA, 2HPMA, 3HPMA AMCC, ATCA, DHBMA and HPMMA) exposure was strongly associated with childhood asthma [21]. In contrast, our study found that the metabolites of 1,3-butadiene, DHBMA and MHBMA3, were not positively associated with the development of asthma in children, but instead may even be present as protective factors. On the other hand, one study found that exposure to toluene and benzene leads to a nearly threefold increase in the incidence of asthma [22]. In contrast, a cross-sectional study showed no statistically significant differences in the levels of formaldehyde, volatile organic compounds, and tobacco-related pollutants between families with and without children with asthma [23].

While the mechanisms by which VOCs increase asthma development are not completely elucidated, there is limited evidence to suggest that early-life exposure to VOCs could cause abnormal activation or overactivation of immune cells and further lead to autoimmune activation [24,25]. Acrolein, the parent compound of CEMA and HPMMA, is a common toxic compound in smoke, is significantly associated with respiratory damage, and can cause smoke inhalation-induced pulmonary edema alone [26]. Additionally, prolonged exposure to acrolein leads to increased mast cell toxicity, increased release of allergenic media, the key components of asthma, and activation of calcium-dependent degranulation pathways [27]. Xylene is the parent compound of 2MHA, which can further induce type 2 helper t cells to participate in the immune response by inducing mast cells and other immune cells to secrete IL4, IL-5, IL-13 and other cytokines [28]. In turn, type 2 helper t cells are the main cells that cause type 1 hypersensitivity reactions such as asthma [29]. Toluene (parent compound of BMA) is thought to cause asthma by activating the IL-6 signaling pathway [30]. However, a study also found no association between urinary 2MHA and BMA concentrations and reduced lung function [20].

There were some limitations of this study. First, NHANES is a cross-sectional survey that limits the ability to establish the temporality of the exposure and outcome sequence, so we could not draw a causal relationship between urinary VOCs metabolites and childhood asthma. Due to the lack of environmental VOCs data, it is not possible to assess environmental exposure to VOCs. Although we observed evidence of an association between 2MHA, BMA and HPMMA and the odds of asthma in some races, there was also a large variability in the number of people per race, which resulted in less precise effect estimates compared to each race. Childhood asthma in this cross-sectional study was sourced from parent-reported questionnaires, and actual asthma was not judged by strict clinical diagnostic criteria, which, together with bias arising from parental recall, may make the actual results deviate significantly from the statistical results. Whereas pulmonary function tests are now available in children aged 5–6 years, the results of pulmonary function tests were missing in the 2013–2018 NHANES cycle, and there remains a need in the future to test mVOCs along with pulmonary function tests in age-appropriate children to ensure that the uncertainty in the actual diagnosis of asthma is reduced. Due to limitations in the survey design of NHANES, there are many potential confounders that we did not have available for model adjustment, such as glomerular filtration rate, parental history of asthma and breastfeeding duration; therefore, there is potential residual confounding from unmeasured or unknown factors.

There are substantial gaps in our knowledge regarding the relationship between mVOCs and asthma risk in children. Although we did not find an overall significant association between mVOCs concentrations in urine and childhood asthma, we did find an association between 2MHA and HPMMA and asthma in female children, and the association between the five mVOCs and asthma varied by race, such that BMA was associated with asthma in non-Hispanic whites and blacks; in Hispanic and non-Hispanic whites, HPMMA was also associated with the development of asthma. Given the pervasive exposure observed among children in the United States, this link warrants further investigation. Further studies could establish prospective cohorts from early infancy to adolescence beforehand and monitor their urinary mVOCs concentrations, immune markers, allergy indicators, and asthma-related indicators such as lung function and identify potential associations that may exist. This will help identify whether there is a period of high susceptibility to asthma risk from long-term VOCs exposure and provide guidance for early life environment improvement and clinical interventions, as well as further enhance our understanding of the possible role of prolonged VOCs exposure in childhood immunity and asthma development.

## 5 Conclusions

Our cross-sectional analysis of a representative sample of US children aged 3–12 years found that urinary VOCs metabolites of acrolein (CEMA and 3PHMA), crotonaldehyde (HPMMA), toluene (BMA), and xylene (2MHA) were associated with childhood asthma, and in particular, 2MHA was significantly associated with the prevalence of asthma in female children. Further studies should establish prospective cohorts of repeated exposures to confirm the causality of the association and to investigate the underlying mechanisms. Studies should also focus on whether the childhood asthma associated with urinary VOCs metabolites found in this study can be prevented by reducing exposure to the corresponding VOCs.

## Funding

This study was supported by grants from the Key Research and Development Program of Sichuan Province (2022YFS0132) and the National Natural Science Foundation of China (82370260).

## Institutional review board statement

Ethical clearance for this study and informed consent of participants were obtained from the NCHS Ethics Review Board, as described on the website: https://www.cdc.gov/nchs/nhanes/irba98.htm.

## Data availability statement

Data associated with this study has been deposited at https://wwwn.cdc.gov/nchs/nhanes/Default.aspx.

## CRediT authorship contribution statement

**Yixiao Xiong:** Writing – original draft, Methodology, Investigation, Data curation, Conceptualization. **Xin Liu:** Validation, Software, Data curation. **Tao Li:** Writing – review & editing, Writing – original draft, Validation, Supervision, Funding acquisition, Conceptualization.

## Declaration of competing interest

The authors declare that they have no known competing financial interests or personal relationships that could have appeared to influence the work reported in this paper.

## Figures and Tables

**Figure:**