# Supplementary material for: The urinary metabolites of volatile organic compounds and asthma in young children: NHANES 2011–2018
Source: Heliyon. 2024 Jan 23;10(3):e24199. doi: 10.1016/j.heliyon.2024.e24199 (PMC10838696; doi:10.1016/j.heliyon.2024.e24199)
Supplement: Supplementary file 1 [file mmc1.docx]

**Supplemental Section**

Title: The urinary metabolites of volatile organic compounds and asthma in young children: NHANES 2011-2018

Authors: Yixiao Xiong^1,2^, Tao Li^1,2*^

Affiliations:

^1^ Department of Anesthesiology, West China Hospital, Sichuan university

^2^ Laboratory of Mitochondria and Metabolism, National-Local Joint Engineering Research Centre of Translational Medicine of Anesthesiology, West China Hospital, Sichuan university

Table S1. URLs of covariates from the NHANES survey in this study.

| Laboratory measurement | Stage | URLs |
| --- | --- | --- |
| Demographic variables | 2011-2012 | https://wwwn.cdc.gov/Nchs/Nhanes/2011-2012/DEMO_G.htm |
|  | 2013-2014 | https://wwwn.cdc.gov/Nchs/Nhanes/2013-2014/DEMO_H.htm |
|  | 2015-2016 | https://wwwn.cdc.gov/Nchs/Nhanes/2015-2016/DEMO_I.htm |
|  | 2017-2018 | https://wwwn.cdc.gov/Nchs/Nhanes/2017-2018/DEMO_J.htm |
| mVOCs | 2011-2012 | https://wwwn.cdc.gov/Nchs/Nhanes/2011-2012/UVOC_G.htm |
|  | 2013-2014 | https://wwwn.cdc.gov/Nchs/Nhanes/2013-2014/UVOC_H.htm |
|  | 2015-2016 | https://wwwn.cdc.gov/Nchs/Nhanes/2015-2016/UVOC_I.htm |
|  | 2017-2018 | https://wwwn.cdc.gov/Nchs/Nhanes/2017-2018/UVOC_J.htm |
| Serum cotinine | 2011-2012 | https://wwwn.cdc.gov/Nchs/Nhanes/2011-2012/COTNAL_G.htm |
|  | 2013-2014 | https://wwwn.cdc.gov/Nchs/Nhanes/2013-2014/COT_H.htm |
|  | 2015-2016 | https://wwwn.cdc.gov/Nchs/Nhanes/2015-2016/COT_I.htm |
|  | 2017-2018 | https://wwwn.cdc.gov/Nchs/Nhanes/2017-2018/COT_J.htm |
| Urine Creatinine | 2011-2012 | https://wwwn.cdc.gov/Nchs/Nhanes/2011-2012/ALB_CR_G.htm |
|  | 2013-2014 | https://wwwn.cdc.gov/Nchs/Nhanes/2013-2014/ALB_CR_H.htm |
|  | 2015-2016 | https://wwwn.cdc.gov/Nchs/Nhanes/2015-2016/ALB_CR_I.htm |
|  | 2017-2018 | https://wwwn.cdc.gov/Nchs/Nhanes/2017-2018/ALB_CR_J.htm |
| Asthma | 2011-2012 | https://wwwn.cdc.gov/Nchs/Nhanes/2011-2012/MCQ_G.htm |
|  | 2013-2014 | https://wwwn.cdc.gov/Nchs/Nhanes/2013-2014/MCQ_H.htm |
|  | 2015-2016 | https://wwwn.cdc.gov/Nchs/Nhanes/2015-2016/MCQ_I.htm |
|  | 2017-2018 | https://wwwn.cdc.gov/Nchs/Nhanes/2017-2018/MCQ_J.htm |

NHANES, the National Health and Examination Survey; mVOCs, metabolite of volatile organic compounds.

Table S2. The detection rates of mVOCs.

| mVOCs | At or above the LLOD | Below LLOD | Missing value |
| --- | --- | --- | --- |
| 2MHA | 2151 (0.740) | 291 (0.100) | 466 (0.160) |
| 34MHA | 2475 (0.851) | 13 (0.004) | 420 (0.145) |
| AAMA | 2471 (0.850) | 3 (0.001) | 434 (0.149) |
| AMCC | 2447 (0.841) | 25 (0.009) | 436 (0.150) |
| ATCA | 2455 (0.844) | 15 (0.005) | 438 (0.151) |
| BMA | 2473 (0.850) | 13 (0.004) | 422 (0.146) |
| BPMA | 1558 (0.536) | 907 (0.312) | 443 (0.152) |
| CEMA | 2460 (0.846) | 23 (0.008) | 425 (0.146) |
| CYMA | 1982 (0.682) | 495 (0.170) | 431 (0.148) |
| DHBMA | 2393 (0.823) | 0 (0.000) | 515 (0.177) |
| GAMA | 933 (0.321) | 1556 (0.535) | 419 (0.144) |
| HEMA | 1387 (0.477) | 1086 (0.373) | 435 (0.150) |
| 2HPMA | 2308 (0.794) | 173 (0.059) | 427 (0.147) |
| 3HPMA | 2418 (0.831) | 8 (0.003) | 482 (0.166) |
| MA | 2420 (0.832) | 49 (0.017) | 439 (0.151) |
| MHBMA3 | 2365 (0.813) | 103 (0.036) | 440 (0.151) |
| PHEMA | 944 (0.325) | 1517 (0.521) | 447 (0.154) |
| PGA | 2467 (0.848) | 12 (0.004) | 429 (0.148) |
| HPMMA | 2487 (0.855) | 0 (0.000) | 421 (0.145) |

Abbreviations: mVOCs: metabolites of volatile organic compound; LLOD: lower limit of detection; 2MHA: 2-methylhippuric acid; 34MHA: 3-and 4-methylhippuric acid; AAMA: N-acetyl-S-(2-carbamoylethyl)-L-cysteine; AMCC: N-acetyl-S-(N-methylcarbamoyl)-L-cysteine; ATCA: 2-aminothiazoline-4-carboxylic acid; BMA: N-acetyl-S-(benzyl)-L-cysteine; BPMA: N-acetyl-S-(n-propyl)-L-cysteine; CEMA: N-acetyl-S-(2-carboxyethyl)-L-cysteine; CYMA: N-acetyl-S-(2-cyanoethyl)-L-cysteine; DHBMA: N-acetyl-S-(3:4-dihydroxybutyl)-L-cysteine; GAMA: N-acetyl-S-(2-carbamoyl-2-hydroxyethyl)-L-cysteine; HEMA: N-acetyl-S-(2-hydroxyethyl)-L-cysteine; 2HPMA: N-acetyl-S-(2-hydroxypropyl)-L-cysteine; 3HPMA: N-acetyl-S-(3-hydroxypropyl)-L-cysteine; MA: mandelic acid; MHBMA3: N-acetyl-S-(4-hydroxy-2-butenyl)-L-cysteine; PHEMA: N-acetyl-S-(phenyl-2-hydroxyethyl)-L-cysteine; PGA: phenylglyoxylic acid; HPMMA: N-acetyl-S-(3-hydroxypropyl-1-methyl)-L-cysteine.

Table S3. Pearson’s Correlation coefficients of differences in current age (y) and age of asthma diagnoses (y) and ln-transformed creatinine-corrected urine mVOCs concentrations among asthmatic children aged 3-12 years (n=246): NHANES (2011-2018).

| mVOCs | CC | *P* |
| --- | --- | --- |
| 2MHA | 0.05 | 0.39 |
| 34MHA | 0.02 | 0.74 |
| AAMA | 0.02 | 0.76 |
| AMCC | -0.12 | 0.06 |
| ATCA | 0.08 | 0.20 |
| BMA | 0.05 | 0.43 |
| BPMA | 0.04 | 0.55 |
| CEMA | 0.02 | 0.72 |
| CYMA | 0.01 | 0.86 |
| DHBMA | 0.02 | 0.75 |
| 2HPMA | -0.02 | 0.74 |
| 3HPMA | -0.05 | 0.44 |
| MA | -0.06 | 0.37 |
| MHBMA3 | 0.03 | 0.61 |
| PGA | -0.03 | 0.61 |
| HPMMA | -0.08 | 0.20 |

Abbreviations: y: years; CC: correlation coefficient; mVOCs: metabolites of volatile organic compounds; NHANES: National Health and Nutrition Examination Survey; 2MHA: 2-methylhippuric acid; 34MHA: 3-and 4-methylhippuric acid; AAMA: N-acetyl-S-(2-carbamoylethyl)-L-cysteine; AMCC: N-acetyl-S-(N-methylcarbamoyl)-L-cysteine; ATCA: 2-aminothiazoline-4-carboxylic acid; BMA: N-acetyl-S-(benzyl)-L-cysteine; BPMA: N-acetyl-S-(n-propyl)-L-cysteine; CEMA: N-acetyl-S-(2-carboxyethyl)-L-cysteine; CYMA: N-acetyl-S-(2-cyanoethyl)-L-cysteine; DHBMA: N-acetyl-S-(3:4-dihydroxybutyl)-L-cysteine; GAMA: N-acetyl-S-(2-carbamoyl-2-hydroxyethyl)-L-cysteine; HEMA: N-acetyl-S-(2-hydroxyethyl)-L-cysteine; 2HPMA: N-acetyl-S-(2-hydroxypropyl)-L-cysteine; 3HPMA: N-acetyl-S-(3-hydroxypropyl)-L-cysteine; MA: mandelic acid; MHBMA3: N-acetyl-S-(4-hydroxy-2-butenyl)-L-cysteine; PHEMA: N-acetyl-S-(phenyl-2-hydroxyethyl)-L-cysteine; PGA: phenylglyoxylic acid; HPMMA: N-acetyl-S-(3-hydroxypropyl-1-methyl)-L-cyst
